# The use of dual antiplatelet therapy for ischemic cerebrovascular events

**DOI:** 10.1007/s10072-022-06395-z

**Published:** 2022-09-17

**Authors:** Francesco Mele, Claudia Gendarini, Leonardo Pantoni

**Affiliations:** 1 Neurology and Stroke Unit, Luigi Sacco Hospital, Milan, Italy; 2 Stroke and Dementia Lab, Department of Biomedical and Clinical Sciences, University of Milan, Via Giovanni Battista Grassi 74, 20157 Milan, Italy

**Keywords:** Minor stroke, TIA, Dual antiplatelet, Intracranial stenosis

## Abstract

In the last 10 years, the use of dual antiplatelet therapy (DAPT) in the neurological ambit has been explored in patients with non-cardioembolic ischemic stroke, transient ischemic attack (TIA), and intracranial atherosclerotic disease. Two clinical trials (CHANCE and POINT) showed that in patients with minor non-cardioembolic ischemic stroke or high-risk TIA, the addition of clopidogrel to aspirin reduces the risk of stroke recurrence. Another trial (THALES) evaluated the association of ticagrelor and aspirin in mild-to-moderate non-cardioembolic ischemic stroke or high-risk TIA, showing a reduced risk of subsequent stroke compared to aspirin alone. Finally, the use of DAPT has been assessed in the treatment of stroke associated with atherosclerotic intracranial stenosis in the SAMMPRIS trial, showing a favorable profile compared to percutaneous angioplasty and stenting. The aim of this article is, after a review the major trials evaluating DAPT in patients with ischemic cerebrovascular events and the ways they have been implemented in Italian, European, and USA guidelines, to provide a practical algorithm to help clinicians in their everyday clinical practice and to outline possible caveats in the practical implementation of guidelines. Possible limitations and gaps in knowledge regarding specific conditions (e.g., the use of DAPT after acute phase therapies) are also underlined.

## Introduction

While the use of dual antiplatelet therapy (DAPT) is well established in cardiology for acute coronary heart disease [1–5], its use in the neurological ambit has been for a long time less proven and only recently has it been established and codified in guidelines.

The need for a proper therapy of ischemic stroke and transient ischemic attack (TIA) derives from the high risk of stroke recurrence. This risk has been estimated around 2% at 12 h, 3% at 2 days, 5% at 7 days, and 10% at 14 days after the acute event [6, 7]. Two pivotal trials, published in 1997, have shown that aspirin is effective in reducing this risk [8, 9]. A Cochrane review has confirmed that the use of aspirin, 160–300 mg daily for 2–4 weeks, reduces the odds of recurrent ischemic stroke by 23% (2.4% aspirin vs. 3.1% control) and those of any recurrent stroke by 12% (3.4% aspirin vs. 3.9% control) [10]. With the aim of further reducing the rate of stroke recurrence, DAPT has received increasing attention in the last years. After one earlier study that was stopped because of the difficulty in recruitment (Fast Assessment of Stroke and Transient ischemic attack to prevent Early Recurrence, FASTER) [11], the current knowledge in this field derives from 4 trials. Three studies have been conducted in patients with minor stroke or TIA: the Clopidogrel in High-Risk Patients with Acute Nondisabling Cerebrovascular Events (CHANCE) study [12], the Platelet-Oriented Inhibition in New TIA and Minor Ischemic Stroke (POINT) study [13], and the Acute Stroke or Transient Ischaemic Attack Treated with Ticagrelor and acetylsalicylic acid for Prevention of Stroke and Death (THALES) study [14]. An additional study has been performed in patients with intracranial atherosclerotic disease: the Stenting and Aggressive Medical Management for Preventing Recurrent Stroke in Intracranial Stenosis (SAMMPRIS) study [15].

The aim of this paper is to briefly review the evidence that has led to establish the role of DAPT in cerebrovascular diseases. We also aimed at synthetizing the approaches of Italian, European, and American guidelines in terms of DAPT indications, showing their similarities and differences. To provide clinicians with an easy-to-read hand-out that integrates the recommendations by the above-mentioned organizations’ guidelines, we also developed an algorithm.

## Methods

We searched PubMed using the query “double antiplatelet therapy AND stroke AND clinical trial” without time period restriction. We also analyzed the most recent guidelines (at the time of submission of the article, July 2022) on ischemic stroke and TIA, from the American Heart Association/American Stroke Association, the European Stroke Organization, and the Italian Stroke Organization. In the guidelines, we looked for any mention of DAPT. The algorithm was developed by integrating the recommendations from these guidelines.

## DAPT in the acute phase of non-cardioembolic TIA and minor ischemic stroke

Three studies have addressed the topic of DAPT in acute TIA or minor-moderate stroke (Table 1). In all of them, aspirin was used, while the second antiplatelet agent was clopidogrel in 2 trials and ticagrelor in the third one.

**Table 1:** Features of the three studies that evaluated DAPT in TIA or minor-moderate stroke

	CHANCE	POINT	THALES
Number of patients (DAPT vs. controls)	5170 (2584 vs. 2586)	4881 (2432 vs. 2449)	11,073 (5523 vs. 5493)
Median age (years)	62	65	65
Inclusion criteria			
Non-cardioembolic ischemic stroke	NIHSS ≤ 3	NIHSS ≤ 3	NIHSS ≤ 5
Non-cardioembolic high-risk TIA	ABCD^2^ score ≥ 4	ABCD^2^ score ≥ 4	ABCD^2^ score ≥ 6
Treatment			
DAPT group	Day 1: Clopidogrel 300 mg + ASA 75–300 mg Day 2–22: Clopidogrel 75 mg + ASA 75 mg Day 22–90: Clopidogrel 75 mg	Day 1: Clopidogrel 600 mg + ASA 50–325 mg Day 2–90: Clopidogrel 75 mg + ASA 50–325 mg	Day 1: Ticagrelor 90 mg × 2 + ASA 300–325 mg Day 2–30: Ticagrelor 90 mg × 2 + ASA 75–100 mg
Control group	Day 1: ASA 75–300 mg + placebo Day 2–90: ASA + placebo	Day 1 – 90: ASA 50–325 mg + placebo	Day 1: ASA 300–325 mg + placebo Day 2–30: ASA 75–100 mg + placebo
Outcomes (DAPT vs. ASA)			
Primary outcome (%)	Stroke at 90 days 8.2 vs. 11.7	Major ischemic event at 90 days 5.0 vs. 6.5	Stroke or death at 30 days 5.4 vs. 6.5
Secondary outcomes			
Ischemic stroke (%)	7.9 vs. 11.4	4.6 vs. 6.3	5.0 vs. 6.2
Severe bleeding (%)	0.2 vs. 0.2	0.9 vs. 0.4	0.5 vs. 0.1

*Abbr. ASA* acetylsalicylic acid, *DAPT* double antiplatelet therapy, *NIHSS* National Institutes of Health Stroke Scale, *TIA* transient ischemic attack

CHANCE was a randomized, double-blind, placebo-controlled trial conducted in 114 centers in China from October 2009 to July 2012 [12]. The study compared the efficacy of DAPT (aspirin plus clopidogrel for 21 days and clopidogrel alone for other 69 days) versus placebo plus aspirin in reducing the risk of recurrent stroke at 90 days in patients with minor ischemic stroke (NIHSS ≤ 3) or high-risk TIA (ABCD^2^ ≥ 4) randomized within 24 h from the event. The results showed that DAPT reduced the occurrence of stroke (event rate 8.2% DAPT vs. 11.7% aspirin, HR 0.68, NNT 29) in particular of ischemic stroke (event rate 8.4% in DATP vs. 11.9% in placebo, HR 0.67) and did not increase the risk of severe bleeding (event rate 0.2% in both groups).

POINT was the second study that evaluated the efficacy of clopidogrel plus aspirin [13]. It was a randomized, international, double-blind, placebo-controlled trial, conducted from May 2010 to December 2017 in 269 sites in 10 different countries (North America, Europe, Australia, and New Zealand), although 82.8% of the patients were from the USA. It randomized 4881 patients within 12 h from non-cardioembolic high-risk TIA (ABCD^2^ ≥ 4) or non-cardioembolic minor ischemic stroke to receive clopidogrel plus aspirin for 90 days or aspirin plus placebo and evaluated the risk of a composite outcome of major ischemic event at 90 days (ischemic stroke, myocardial infarction, death due to an ischemic event). The study was stopped in August 2017 because the major bleeding boundary was exceeded. At that time, major ischemic event occurred less frequently in patients treated with DAPT (event rate 5.0% DAPT vs. 6.5% aspirin, HR 0.75, NNT 66), and the occurrence of ischemic stroke was reduced in the treatment group (event rate 4.6% DAPT vs. 6.3% aspirin, HR 0.72), although the risk of major bleeding was significantly increased (event rate 0.9% DAPT, vs. 0.4% aspirin, HR 2.32, NNH 200).

The third trial was the THALES, a randomized, placebo-controlled, double-blind trial conducted in 414 sites in 28 countries from January 2018 to October 2019 [14]. It enrolled 11,073 patients with non-cardioembolic high-risk TIA (ABCD^2^ ≥ 6) or mild-to-moderate non-cardioembolic ischemic stroke (NIHSS ≤ 5) within 24 h from onset and evaluated the efficacy of ticagrelor plus aspirin in reduction of the risk of subsequent stroke or death at 30 days. Five thousand five hundred twenty-three patients were assigned to the treatment group (ticagrelor + aspirin) and 5493 to the aspirin group (aspirin + placebo). The occurrence of the primary outcome was significantly reduced in the DAPT group (event rate 5.4% DAPT, 6.5% aspirin, HR 0.83, NNT 92), and so was the occurrence of ischemic stroke (event rate 5.0% DAPT, 6.2% aspirin, HR 0.79). Considering safety, severe bleeding risk was significantly increased in the treatment group (event rate 0.5% DAPT, 0.1% aspirin, HR 3.99, NNH 263), and intracranial hemorrhages or fatal bleedings were more frequent in patients treated with DAPT (event rate 0.4% DAPT, 0.1% aspirin, HR 3.66).

In conclusion, the three above-mentioned studies show a slight but statistically significant favorable effect of DAPT in patients with minor ischemic stroke or high-risk TIA. Although the NNT values are quite high (especially for the association of aspirin and ticagrelor), the use of DAPT in these patients is supported by the high incidence rate of minor stroke and TIA in the overall population, inducing a net benefit in a consistent number of patients.

## DAPT in patients with stroke due to intracranial stenosis

The use of DAPT has been considered also for the treatment of atherosclerotic intracranial stenosis. SAMMPRIS was an investigator-initiated, randomized, clinical trial conducted in 50 sites in the USA from November 2008 to April 2011 [15]. It enrolled 764 patients that had a TIA or a non-disabling stroke, within 30 days of acute event, attributable to a stenosis of 70–99% of a major intracranial artery. The aim of the study was to compare the efficacy of percutaneous transluminal angioplasty and stenting (PTAS) plus aggressive medical therapy versus aggressive medical therapy alone in the treatment of symptomatic intracranial stenosis. Aggressive medical therapy consisted of aspirin 325 mg/die plus clopidogrel 75 mg/die for 90 days in addition to management of primary and secondary risk factor (elevated systolic blood pressure, elevated LDL and non-HDL cholesterol levels, diabetes, smoking, excess of weight, and insufficient exercise). PTAS procedure was performed within 3 business days after randomization by an experienced neurointerventionist. Patients have been followed for at least 1 year, and the primary endpoints were stroke or death within 30 days after enrollment or after revascularization procedure or ischemic stroke in the territory of the symptomatic artery after day 30 and the end of follow-up. The enrollment was stopped in 2011 because a futility analysis suggested that there was no benefit from PTAS while the risk of periprocedural stroke or death was increased. At the time of interruption, the occurrence of the primary outcome was significantly lower in the medical therapy group (event rate 11.5%, probability of events at 30 days 5.8% and probability at 1 year 20.0% in medical group vs. event rate 20.5%, probability of events at 30 days 14.7%, probability at 1 year 12.2 in PTAS group; *p* = 0.009); the difference was primarily due to different rate of ischemic stroke within 30 days (4.4% medical vs. 10.3% PTAS) and of symptomatic brain hemorrhage within 30 days (0 medical vs. 4.5% PTAS). Considering safety outcomes, major hemorrhage was significantly more frequent in patients treated with medical therapy plus PTAS (event rate 2.2%, probability of events at 30 days 0.9%, and probability at 1 year 1.8% in medical group vs. event rate 9.8%, probability of events at 30 days 8.0%, probability at 1 year 9.0 in PTAS group; *p* < 0.001).

We feel this evidence is of particular importance since the amelioration and diffusion of imaging techniques for the study of intracranial atherosclerosis are showing that this is a relevant stroke mechanism in many patients, conferring one of the greatest risks of stroke recurrence [16]. The SAMMPRIS study therefore clarifies that in this relevant etiologic group of stroke patients, a tailored approach based on DAPT represents the most effective choice for secondary prevention.

## DAPT for cerebrovascular events according to current guidelines

The above-reported evidence has been the basis for the formulation of guidelines released by national and international organizations and associations.

The Italian Stroke Prevention and Educational Awareness Diffusion (SPREAD) [17], the European Stroke Association (ESO) [18], and the American Heart Association/American Stroke Association (AHA/ASA) [19] strongly recommend the use of DAPT with aspirin and clopidogrel, followed by single antiplatelet therapy, in case of non-cardioembolic minor ischemic stroke (NIHSS score of 3 or less) or high-risk TIA (ABCD^2^ score of 4 or more). The differences across these recommendations are in the suggested duration of DAPT (30 days for SPREAD, 21 days for ESO, 21–90 for AHA/ASA) and in the interval between the event and the introduction of therapy (24 h for ESO, ideally 12–24 h and at least within 7 days for AHA/ASA). Moreover, the SPREAD guidelines suggest considering at high risk a TIA not only if the ABCD^2^ score is ≥ 4, but also if the patient has an intracranial stenosis or evidence of microembolism for a carotid plaque at transcranial Doppler.

ESO and AHA/ASA also consider the use of DAPT with aspirin and ticagrelor for 30 days followed by single antiplatelet therapy in patients with non-cardioembolic, mild-to-moderate, ischemic stroke (NIHSS of 5 or less) or high-risk TIA (ABCD^2^ score of ≥ 6 or other high-risk features) in the past 24 h. High-risk features are defined as intracranial atherosclerotic disease or at least 50% stenosis in an internal carotid artery that could account for the event. Both guidelines consider the quality of evidence for this treatment moderate and therefore release a weak strength recommendation for intervention.

Finally, AHA/ASA and ESO suggest the use of DAPT with aspirin and clopidogrel for 90 days in patients with recent stroke or TIA (within 30 days) attributable to severe stenosis (70–99%) of a major intracranial artery, with a strength of recommendation that is defined moderate by the AHA/ASA and weak by the ESO [19, 20].

Table 2 highlights the differences across these three guidelines.

**Table 2:** Differences in indications for DAPT across guidelines

	AHA/ASA	ESO	SPREAD
Aspirin + clopidogrel in non-cardioembolic ischemic stroke and high-risk TIA			
Patients	- Non-cardioembolic minor ischemic stroke (NIHSS ≤ 3) - High-risk TIA (ABCD^2^ ≥ 4)	- Non-cardioembolic minor ischemic stroke (NIHSS ≤ 3) - High-risk TIA (ABCD^2^ ≥ 4)	- Non-cardioembolic minor ischemic stroke (NIHSS ≤ 3) - High-risk TIA (ABCD^2^ ≥ 4 or intracranial stenosis or microembolism at TCD)
Time from event to start of DAPT	Ideally 12–24 h, at least 7 days	24 h	Possibly, 12 h
Duration of DAPT	21–90 days	21 days	30 days
Aspirin + ticagrelor in non-cardioembolic ischemic stroke and high-risk TIA			
Patients	- Minor to moderate stroke (NIHSS ≤ 5) - High-risk TIA (ABCD^2^ ≥ 6 OR symptomatic intracranial or extracranial ≥ 30% stenosis of an artery that could account for the event)	- Non-cardioembolic mild to moderate ischemic stroke (NIHSS ≤ 5) - High-risk TIA (ABCD^2^ ≥ 6 OR high-risk features: either intracranial atherosclerotic disease or at least 50% stenosis in an internal carotid artery that could account for the presentation)	No specific indication-
Time from event	24 h	24 h	
Duration of DAPT	30 days	30 days	
DAPT in stroke and TIA due to intracranial stenosis			
Therapy	Aspirin + clopidogrel	DAPT (aspirin + cilostazol or clopidogrel or ticagrelor)	Aspirin + clopidogrel
Patients	- Stroke attributable to severe stenosis (70%–99%) of a major intracranial artery - TIA attributable to severe stenosis (70–99%) of a major intracranial artery	- Ischemic stroke related to intracranial stenosis due to ICAD - TIA related to intracranial stenosis due to ICAD	- Minor ischemic stroke associated with intracranial stenosis - TIA associated with intracranial stenosis
Time from event	30 days	No specific indication-	No specific indication
Duration of DAPT	90 days	90 days	90 days

## Proposal of an algorithm for the use of DAPT in acute ischemic stroke and TIA

**Fig. 1:**
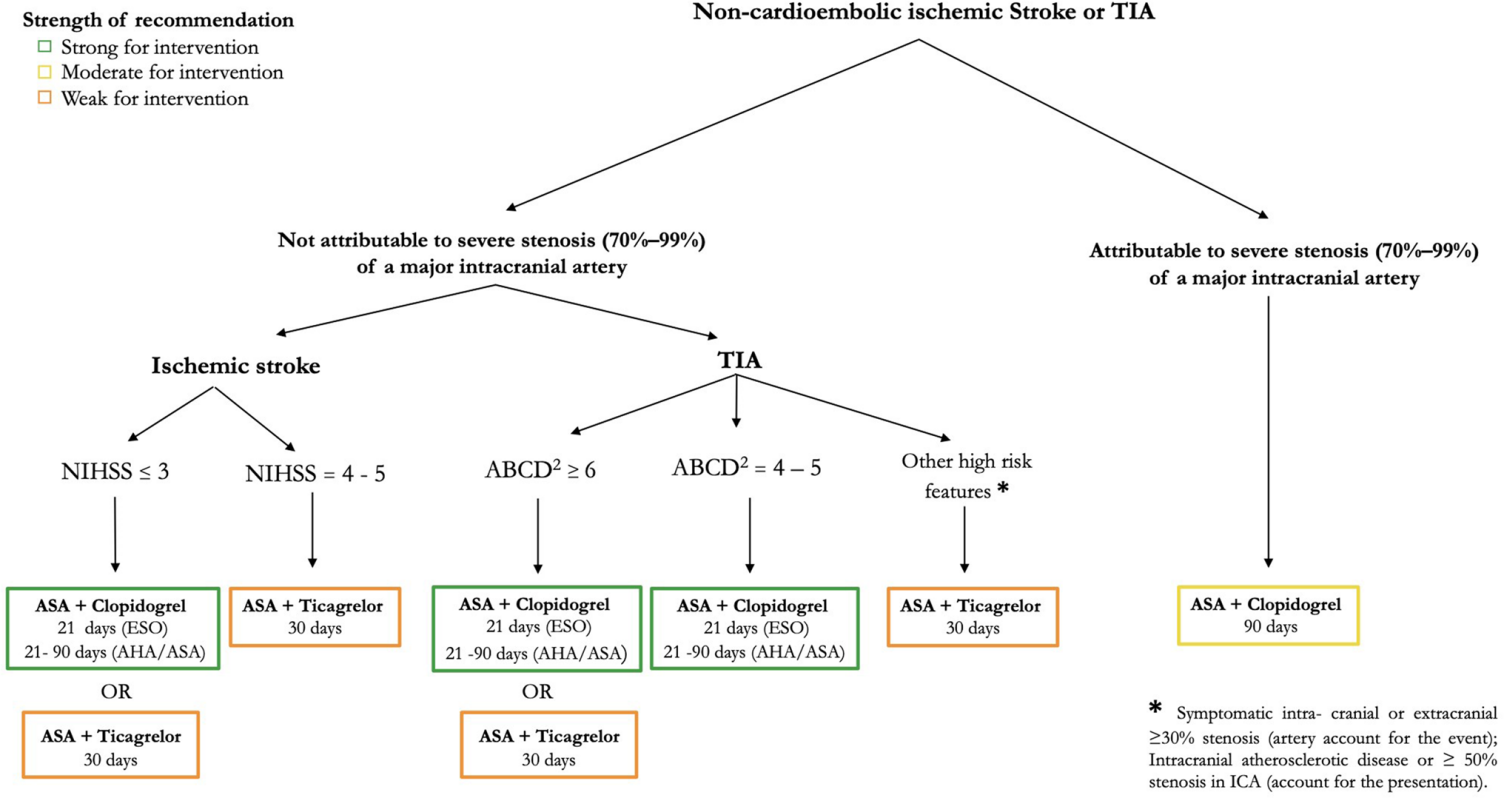
Proposed algorithm: summarizing current guidelines on the use of DAPT. AHA/ASA, American Heart Association/American Stroke Association; ASA, acetylsalicylic acid; DAPT, double antiplatelet therapy; ICA, internal carotid artery; NIHSS, National Institutes of Health Stroke Scale; TIA, transient ischemic attack

To help clinicians to follow the recommendations and to integrate some discrepancies across guidelines, we propose the algorithm that is reported in Fig. 1. The use of DAPT in the field of cerebrovascular disease remains limited to few conditions and is intended for a restricted time frame. Some limitations should also be considered due to a lack of evidence in some specific conditions.

All studies and guidelines presented in this review grade the severity of stroke considering only the NIHSS score, without evaluating the dimension of the lesion. Since the NIHSS strongly depends on the motor function and language deficits, there is a good correlation between the score and the lesion size in case of anterior circulation strokes but, in case of posterior circulation strokes, NIHSS can underestimate the ischemic lesion size [21–23]. A typical case is that of a posterior cerebral artery lesion that can be associated with a very low NIHSS but nonetheless be of a considerable size. In clinical practice, the true size of the lesion can be difficult to appreciate within the time frame for the initiation of DAPT proposed by some guidelines (i.e., 12 h) unless an MRI is used. The implementation of an MRI for each patient clinically eligible for DAPT can be troublesome on a large scale. Considering the above reported cases, we suggest that in case of acute isolated visual field deficits, the introduction of DAPT should be postponed of 24 h if an MRI is not obtainable within the first 12 h and be initiated after the execution of a second CT at 24-h interval.

Another point that has remained unanswered in clinical trials, and therefore is without a clear indication in guidelines, is the use of DAPT in patients who have received an acute-phase treatment (intravenous thrombolysis, mechanical thrombectomy, or both). In the POINT study [13], patients who were candidate for thrombolysis or endovascular intervention were excluded; in THALES [14], thrombolysis and mechanical thrombectomy within 24 h before the randomization were exclusion criteria. CHANCE did not have specific exclusion criteria for thrombolysis, but none of the included patients was treated with thrombolysis [12]. Thus, there is no current evidence of the efficacy and safety of DAPT in patients treated with thrombolysis or mechanical thrombectomy.

## Conclusions

From the literature review, the use of DAPT in stroke patients appears safe and useful in at least two acute conditions, i.e., non-cardioembolic minor stroke/TIA and stroke due to intracranial stenosis. At present, evidence for other indications is lacking except those in which the DAPT requirement is set by the positioning of a stent. According to the Italian and international guidelines, DAPT should be started early after the event and continued for limited time periods. Because some minor differences exist across guidelines and utilized drugs, the proposed algorithm tries to overcome some of the difficulties that clinicians may encounter when facing a patient in the first few hours following an acute ischemic cerebrovascular event, for example in the choice of the most appropriate drugs. Future studies will clarify whether the benefit obtained by DAPT is sustained over time and whether the implementation of current guidelines is effective in clinical practice.
